# Open group dialogue on post-mortem organ donation promotes attitudinal change among different groups of the Italian population

**DOI:** 10.3389/fpsyg.2025.1631504

**Published:** 2025-10-14

**Authors:** Silvia Caterina Maria Tomaino, Francesco Procaccio, Teresa Armanni, Massimo Cardillo, Paola Di Ciaccio, Margherita Gentile, Sabrina Cipolletta

**Affiliations:** ^1^Department of General Psychology, University of Padua, Padua, Italy; ^2^Centro Nazionale Trapianti, Istituto Superiore di Sanità (ISS), Rome, Italy

**Keywords:** attitude, organ donation, health knowledge, tissue and organ procurement, organ procurement organization

## Abstract

**Introduction:**

A high discrepancy between a generally positive attitude and consent to donation has been observed in Italy, as in 2021 only 68.2% of registered individuals had provided consent. Understanding which variables may play a role in this decision-making process, considering the experiences of different groups, is essential to support the development and implementation of targeted policies. The aim of this study was to investigate the demographic and psychosocial variables associated with the decision to register consent for post-mortem organ donation in Italy, and to compare the experiences of different population groups to support the development of targeted policies.

**Methods:**

A quantitative study was conducted in 2021 in collaboration with the National Centre for Transplantation. A total of 353 participants–including healthcare professionals, citizens, opinion leaders and registry office employees–completed an ad-hoc questionnaire before and after participating in a focus group on organ donation. Descriptive statistics and regression analyses were conducted.

**Results:**

Of the 353 participants, 93.8% reported a positive attitude toward post-mortem organ donation (score > 5 on a 7-point Likert scale). In the pre-focus group questionnaire, the mean attitude was 6.45 (SD = 1.05), which increased significantly to 6.56 (SD = 0.99) after the focus groups (*Z* = −4.06, *p* < 0.001). Regarding actual behavior, 50.4% had already registered their consent to donation. Significant associations emerged between positive attitude and gender (women reporting higher scores; *U* = 13,129, *p* = 0.045), level of education (*r* = 0.156, *p* = 0.004), familiarity with donation (e.g., knowing a donor or someone who registered consent; *p* < 0.001), and being registered with donation-related associations (*p* < 0.001). Intention to register was strongly predicted by attitude (*p* < 0.001), and actual consent registration was more likely among participants with higher education and those familiar with donation practices.

**Conclusion:**

Findings highlight the role of demographic factors, familiarity, and personal values in shaping donation behavior, supporting the use of multivariable models to better explain consent registration. These insights underline the need to implement targeted awareness campaigns and policies aimed at promoting informed choices about organ donation.

## 1 Introduction

Organ donation represents a crucial medical and social issue worldwide, as it is the only therapeutic option for many patients affected by end-stage organ failure. Advances in transplantation have significantly improved survival and quality of life; however, the persistent shortage of donors remains a major challenge for health systems, leading to long waiting lists and, in many cases, preventable deaths. According to international reports (European Parliament, 2018), the demand for organs continues to outpace supply, with thousands of patients each year unable to access a life-saving transplant. This shortage underlines the need to better understand the determinants of individuals’ willingness to donate and to develop effective strategies to promote informed decisions and increase the number of registered consents. Law regulations about post-mortem organ donation vary, depending on the country, with each implementing a complex legislative and regulatory systems that could face difficulties in their complete application, potentially causing disruptions for services and citizens, resulting in the frequent implementation of mixed systems or their partial application.

In Italy organ donation is regulated by Law n°91/99 that specifies an “opt-out” system of presumed consent that establishes that a citizen is considered a post-mortem donor unless they explicitly oppose this during their lifetime. A similar system is implemented in Spain, Austria and France as opposed to the “opt-in” system involving explicit consent, that is implemented, for example, in Denmark, Germany and Ireland (European Parliament, 2018). To date, Law n°91/99 has not been fully implemented in Italy, causing general disruption and low rates of donation, and actually resulting in more than 8,000 Italian citizens being held on a waiting list to receive organ transplants (Centro Nazionale Trapianti, 2022). To register one’s consent to donation regarding post-mortem organ donation in Italy, there are different available modalities such as registering with Local Health Units (LHU), registering with the Italian Association of Organ Donors (AIDO), registering for the *donor card* of the Ministry of Health, redacting an autographed and dated piece stating one’s consent about donation to include in one’s documents, and registering one’s consent to donation during the procedure of electronic identity card (CIE) renewal at one’s Municipality. In 2021, the most frequent modality of registration was the CIE (86.6%) followed by AIDO (11.8%) and LHU (1.6%); a total of 3,201,540 choices about donation have been registered, of which 2,204,318 were consent to donation and 997,222 were in opposition (Centro Nazionale Trapianti, 2021). In 2021, out of the total population over 18 years of age, the percentage of those who registered their choice with regard to organ donation was 63.5%, underlining the high number of abstentions.

When defining the behavior of post-mortem organ donation, three main constructs are taken into consideration: attitude, intention and registration as a donor (Falomir-Pichastor et al., 2011).

Even though the majority of the population reports a positive attitude towards organ donation (Boulware et al., 2002; Brug et al., 2000; Moloney et al., 2019; Morgan et al., 2008; Rumsey et al., 2003) this does not necessarily result in an effective and registered consent to donation (Brug et al., 2000; Moloney et al., 2019; Morgan et al., 2008), pointing out the need for a further exploration of the variables that could play a role in determining this discrepancy.

The decision to become an organ donor appears as a complex choice that can be negatively influenced by different aspects which are both personally, socially and culturally determined, such as fear and doubts regarding death, and the comprehension of brain death (Skowronski et al., 2020), the integrity of the donor’s body (Lauri, 2009; Miller et al., 2020), general misinformation (Arisal and Atalar, 2019; Lomero et al., 2017), religious beliefs (Lauri, 2009; Moloney and Walker, 2002) and lack of trust in the healthcare system (Miller et al., 2020).

On the other hand, variables that are associated with a positive attitude towards organ donation are being a female (Stadlbauer et al., 2020), being between 30 to 50 years of age and feeling socially responsible for one’s community (Falomir-Pichastor et al., 2011), having suffered a long illness (Mossialos et al., 2008), being a blood donor (Cossé and Weisenberger, 2000; Hyde et al., 2013) and being familiar with the topic in terms of knowing someone who has received/donated, or is waiting to receive, an organ (Caballer et al., 2000).

The Theory of the Planned Behavior (TPB) suggests that the intention of a person to engage in a certain behavior can be predicted by their intention to engage in that behavior (Ajzen, 1991). This theoretical framework has already been applied to the field of organ donation to explain how personal beliefs and social factors influence the intention to become a donor and the registration of consent (Pauli et al., 2017; Siegel et al., 2008). According to this model, personal beliefs about organ donation play a role in the determination of attitude towards organ donation and intention to become a donor, pointing out that these intentions strongly predict an explicit consent to donate. In our study, the TPB was adopted as a theoretical reference to guide the selection of variables (attitude, intention, consent registration) and to interpret the relationships among them. This study was conducted concurrently with and in-depth qualitative exploration of perceptions, beliefs and information around organ donation and consent registration, based on the qualitative analysis of focus group discussions, the results of which are presented in Cipolletta et al. (2023). The specific application of TPB-based regression models is presented in the Methods section.

In this sense, exploring attitudes from different perspectives is of great importance when it comes to understanding the needs and experiences of the different actors involved in the process of donation and consent registration. To date, many studies (Canova et al., 2006; Fontana et al., 2017; Terraneo and Caserini, 2022) have explored the points of view of healthcare professionals and students in Italy as well as in other countries (Elsafi et al., 2017; Hakeem et al., 2021), while, to our knowledge, few have taken into consideration the point of view of the general population (Cohen and Hoffner, 2012; Falomir-Pichastor et al., 2011; Lauri, 2009) and none the one of professionals involved in the process of consent to donation registration (e.g., registry office employees).

The aim of the present study originated from the evidence of a still low percentage of consent to donation registrations in Italy, together with the discrepancy between a general positive attitude and the effective number of positive registered consents, and from the importance of taking into account that the choice of becoming a post-mortem organ donor and the registration of one’s consent to donation involves many personal, social and cultural aspects as well as services and people. Starting from this premise, the present study aims to explore the attitude, intention to donate and consent to donation in different groups of the Italian population, aiming to provide specific knowledge regarding the different groups involved. In this sense, this is the first study to consider different population groups that are part of this process, in the form of those required to make their choice (citizens), those required to ask and register the consent to donation (registry office employees), and those who have a key role in the process of decision making on a practical (healthcare professionals) and community (opinion leaders) level. Results gained from the present study could foster and support the importance of including the perspective and framework of social psychology in the investigation of this topic as well as in the implementation of the deriving policies. In fact, providing institutions and policy makers with knowledge about the specific populations involved in this process and about the social influences playing a role in the decision of becoming a post-mortem organ donor is of great importance to improve local and international policies and intervention, as well as organ to reduce donation recipients’ waiting lists.

## 2 Materials and methods

### 2.1 Research design and participants

A total of 353 participants took part in the mixed-method research carried out between the 1^st^ June and 30th November 2021 (mean age of 45.45, range of 18–77). This involved participation in focus groups (data not presented in this paper) with regard to post-mortem organ donation and consent registration, and in the completion of pre-post questionnaires. Out of the total of the sample, 144 (40.8%) were male, 208 (58.9%) female, and 1 participant (0.3%) did not specify gender. Regarding civil status, the majority were married or cohabitant (202, 57.2%), followed by single participants (98, 27.8%). Smaller proportions reported being divorced (14, 4%), separated (14, 4%), or widowed (13, 3.7%). Educational attainment was heterogeneous: 19 participants (5.4%) had a middle school diploma, 35 (9.9%) a high school diploma, 49 (13.9%) a technical school diploma, 46 (13.0%) a bachelor’s degree, 84 (23.8%) a master’s degree, and 105 (29.8%) postgraduate education; 9 (2.5%) reported other qualifications, and 6 (1.7%) did not specify.

Participants came from different Italian regions, with higher representation from Campania (80; 22.7%), Piedmont (78; 22.1%), and Puglia (55; 15.6%), followed by Abruzzo (47; 13.3%), Lombardy (47; 13.3%), and Tuscany (46; 13%). Participants were grouped based on inclusion criteria, their characteristics and group composition are reported in Table 1.

**Table 1:** Participants’ characteristics and groups compositions.

**Gender**	***N***	**%**	**Civil status**	***N***	**%**	**Education**	***N***	**%**	**Region of origin**	***N***	**%**	**Group of belonging**	**Groups’ number**	***N***	**%**
Male	144	40.8%	Married or Cohabitant	202	57.2%	Middle School diploma	19	5.4%	Tuscany	46	13.0%	Local healthcare professionals	5	55	15.6%
Female	208	58.9%	Widowed	13	3.7%	High school diploma	35	9.9%	Abruzzo	47	13.3%	Critical area healthcare professionals	6	56	15.9%
Not specified	1	0.3%	Divorced	14	4.0%	Technical school diploma	49	13.9%	Piedmont	78	22.1%	Young adult population	6	55	15.6%
			Separated	14	4.0%	Bachelor’s degree	46	13.0%	Campania	80	22.7%	Adult population	6	57	16.1%
			Single	98	27.8%	Master’s degree	84	23.8%	Lombardy	47	13.3%	Opinion leaders	4	27	7.6%
			Total	341	96.5%	Postgraduate	105	29.8%	Puglia	55	15.6%	Registry office employees	6	48	13.6%
			Missing	12	3.4%	Other	9	2.5%				Hospital Healthcare professionals	5	55	15.6%
						Not specified	6	1.7%							

### 2.2 Inclusion and exclusion criteria

The participants recruited were identified in terms of the following groups and inclusion criteria:

1. Young adult population: being an Italian citizen, between 18 and 40 years of age.
2. Adult population: being an Italian citizen, between 41 and 80 years of age.
3. Registry office employees: being involved in the consent to donation registration process in their municipality.
4. Hospital healthcare professionals: working in a hospital context but not directly involved in the donation and/or transplantation departments and processes.
5. Critical area healthcare professionals: working in Intensive Care Units (ICU) and/or other departments specifically involved in the donation and/or transplantation process.
6. Local healthcare professionals: working as general practitioners, family doctors or in the local clinics.
7. Opinion leaders: being a social and community influential person such as municipal councilors, teachers, priests, religious leaders, social media influencers with more than 500 k followers, journalists and more.

The participants were recruited by telephone, e-mail and personal approach, primarily through the professionals of each Regional Transplant Centers. Through the personal and professional networks of the people working at the CRTs, lists of individuals belonging to different research groups were identified and compiled.

Specifically, healthcare professionals were recruited through colleagues working in hospitals and across the local healthcare system. For the younger population, connections were established with schools, universities, and community associations. Engagement with opinion leaders was facilitated through collaboration with the press offices of hospitals and the relevant regional authorities. Furthermore, with regard to registry office employees, the CNT and the CRTs hold a comprehensive mapping of contacts and designated representatives within each registry office. All participants received an invitation letter and an informed consent form with regard to participation in the study and data processing, the informed consent was obtained in written form. Exclusion criteria were being a living donor or an organ recipient, to ensure that the focus group discussion and the responses to the pre-post questionnaires were not influenced.

### 2.3 Measures

Data analyzed in this paper have been collected with a pre-post questionnaire constructed *ad hoc* by the researchers that the participants completed using pen and paper, both before the start of the focus group, and immediately after its conclusion.

Data were collected anonymously (each participant created an alphanumerical code to help researchers associate pre-post responses without exposing personal data).

The pre-focus group questionnaire required 5 min for completion and was composed of two parts: demographic information and knowledge, attitude and intention about donation and consent to donation. The post-focus group questionnaire required 8–10 min for completion and was composed of two parts: knowledge, attitude, intention and consent to donation registration, and evaluation of the participation in the focus group.

### 2.4 Data analyses

Data were analyzed with the use of SPSS. The main dependent variables were attitude toward post-mortem organ donation (measured on a Likert scale), perceived importance of donation, intention to register consent, and actual consent registration. Independent variables included demographic characteristics (age, gender, education, geographical area), familiarity with organ donation (e.g., knowing a donor/recipient or someone who had registered consent), registration with donation-related associations, and group membership (general population, healthcare professionals, opinion leaders).

Associations between demographic variables and continuous outcomes were tested using Spearman’s rank correlation. Differences in attitude and perceived importance between groups were examined with Mann–Whitney *U* tests for two groups and Kruskal–Wallis tests for three or more groups. Group differences in categorical outcomes such as consent registration and intention were analyzed using chi-square tests. Pre–post variations in attitude, importance, and intention following focus group participation were assessed with Wilcoxon signed-rank tests. Logistic regression was employed to test whether predictors including attitude, importance, intention, demographic factors, and familiarity explained the likelihood of actual consent registration, with odds ratios and 95% confidence intervals reported. Finally, linear regression was used to test the Theory of Planned Behavior model, with attitude as the main outcome and demographics, familiarity, and prior reflection on donation as predictors; subsequent models examined whether attitude and importance predicted intention and consent registration.

### 2.5 Ethics

The study was conducted in accordance with the Declaration of Helsinki and approved by the Ethics Committee of the School of Psychology of the University of Padua, Italy (protocol 3,749, approved on 19 October 2020).

## 3 Results

### 3.1 Direct experience with the topic of post-mortem organ donation

Participants were asked “Have you ever thought of donating your organs after death?.” Out of the total, 85.5% answered yes, 10.8% no and 1.7% I do not know.

In Table 2 are reported the questions asked and the results regarding the participants’ familiarity with the topic of post-mortem organ donation. A question explored the registration with associations related to the topic of organ donation (such as donation of blood, organs, bone marrow), showing that 95 (26.9%) were registered to at least one.

**Table 2:** Participants’ familiarity with the topic of organ donation and consent to donation.

Question	Yes		No		Not sure	
	*N*	%	*N*	%	*N*	%
Have you got a friend/acquaintance/relative who received an organ?	115	32.6	224	63.5	12	4
Have you got a friend/ acquaintance/relative who donated an organ?	39	11	269	76.2	45	12.7
Have you got a friend/acquaintance/relative who registered their consent to post-mortem organ donation?	180	51	111	31.4	62	17.6

### 3.2 Attitude toward post-mortem organ donation

The majority of the participants - 331 (93.8%) - reported a high score in terms of their attitude toward post-mortem donation (score > 5 on a Likert scale of 1–7). In the pre-focus group questionnaire the mean attitude reported was 6.45 (SD = 1.05), compared to the post-focus group questionnaire when the mean attitude was 6.56 (SD = 0.99). In the pre-focus group questionnaires, 168 participants (47.6%) stated donation as being “essential,” 180 (51%) as “important” and 5 (1.4%) did not respond on a scale (essential, important, not important and useless).

The post-focus group questionnaire assessed the intention to donate one’s organs after death (see Table 3). Participants were asked if they had already registered their consent regarding post-mortem organ donation regardless of their decision. Out of the total, 178 participants (50.4%) reported having registered their consent to donation already, while 169 (47.9%) did not and 6 (1.7%) did not answer.

**Table 3:** Participants’ intention to register one’s choice about donation and to register one’s consent.

Question	Yes		No		I do not know	
	*N*	%	*N*	%	*N*	%
“Would you register your choice about donation?”	307	92.2	13	3.9	13	3.9
“Would you give your permission to the post-mortem organ donation?”	309	87.5	17	4.8	27	7.6

### 3.3 Variables influencing attitude

**Figure 1:**
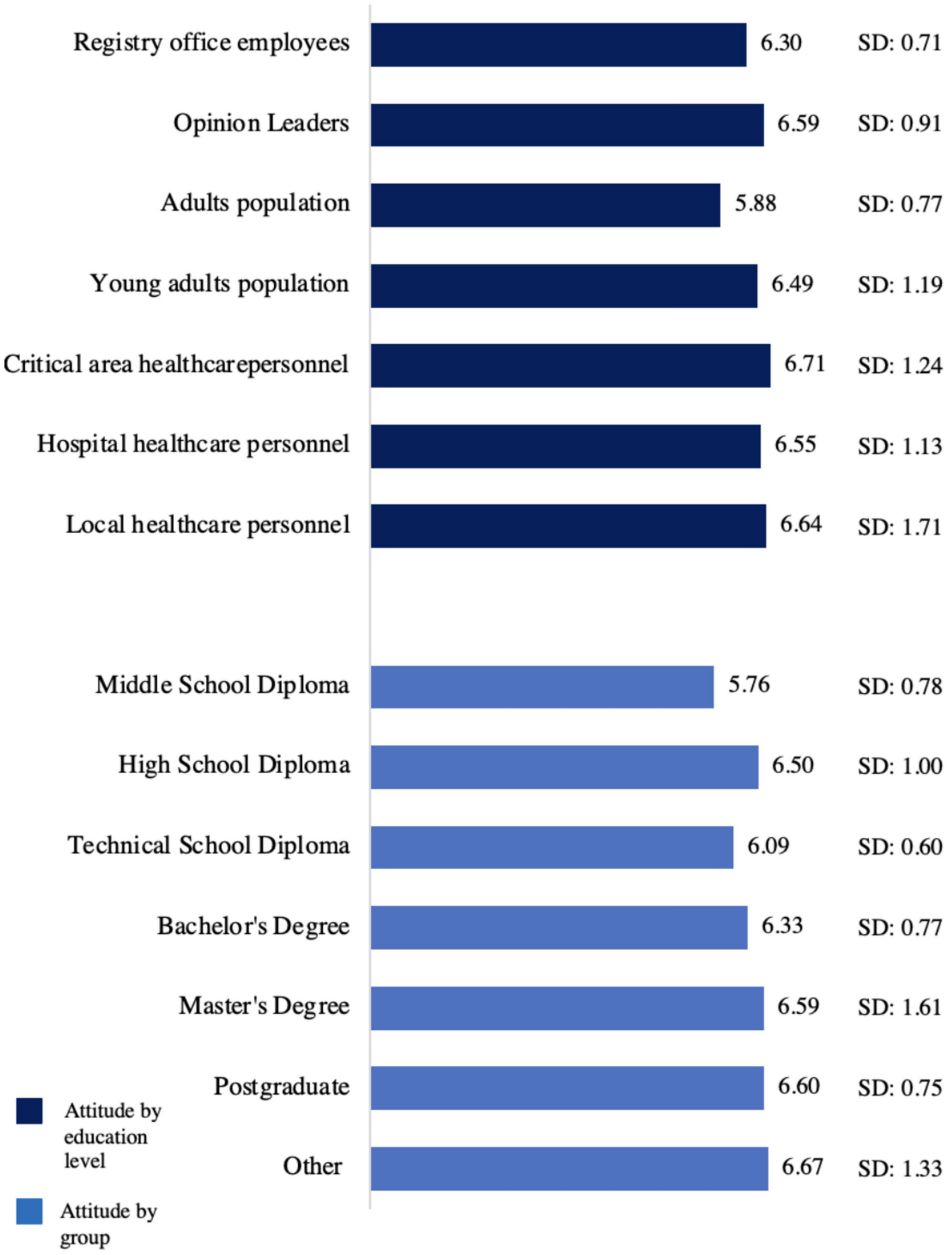
Mean attitude per group of belonging and education level.

A significant correlation was found between age and the perceived importance of donation (*r =* −0.15; *p* = 0.006), but not with attitude (*r =* −0.09; *p* = 0.07), nor with consent registration (*U* = 14490.5, *p* = 0.902). Out of the sample total, 70 men (49%) and 108 women (53.2%) had already registered their consent to donation. Women showed a significantly more positive attitude towards post-mortem donation (*U* = 13,129; *p* = 0.045) compared to men; no significant association was shown between gender and perceived importance of donation (*U* = 14216.5, *p* = 0.616), as well as consent to donation [χ^2^(2) = 0.607, *p* = 0.447]. Education was significantly correlated with attitude (*r = 0*.156, *p* = 0.004) and importance of post-mortem donation (*r = 0.*169, *p* = 0.002). Furthermore, a positive albeit non-significant relationship (*p* = 0.186) was found (U = 12727.5, z = −1.32) between level of education and consent to donation. Differences in mean attitude per level of education are reported in Figure 1.

No differences in attitude and importance with regard to post-mortem donation were found in terms of geographical area; North mean score 6.54 (SD = 0.9), Center 6.37 (SD = 1.18) and South 6.42 (SD = 1.08). No differences regarding the importance of donation and geographical area were found (*H* = 0.73, *p* = 0.695). Moreover, differences in the consent to donation registration rate were found, even though they were non-significant [χ^2^(2) = 4.296, *p* = 0.117]. The 58.1% of respondents from the North reported having registered their consent to donation, followed by participants from the South (50%) and the Center (44.1%).

Out of the total sample, 97 participants (27.48%) reported being registered with at least one association regarding organ donation. This was not significantly associated with attitude towards donation (*p* = 0.07), but rather was associated with consent to donation registration (*p* < 0.001). In this sense a person registered to an association with regard to donation was more likely to have registered their consent to post-mortem organ donation.

Knowing someone who had donated or who had received an organ was associated with a more positive attitude towards organ donation (*U* = 11,530.5, *p* < 0.001) and with a higher importance attributed to the topic (*U* = 12,304, *p* = 0.023). Knowing someone who had registered their consent to donation was also associated with attitude towards donation (*U* = 10,553, *p* < 0.001) and its importance (*U* = 12,318, *p* = 0.001). Furthermore, such participants were more likely to have registered their own consent to donation [χ^2^(1) = 153.65, *p* < 0.001]. Mean attitude and importance in the familiarized and non-familiarized groups is shown in Table 4. Mean attitude variations based on the group of the Italian population and on the education level are shown in Figure 1. Choice about donation registration varied in terms of the group under consideration (see Table 5). Specifically, the group that reported the highest choice to donation registration was that of hospital healthcare professionals (67.3%), while the one that reported the lowest percentage was that of the opinion leaders (38.5%).

**Table 4:** Mean attitude and importance in the familiarized and non-familiarized groups.

Familiarity	Yes		No		Yes		No	
	Mean attitude	SD	Mean attitude	SD	Mean importance attributed	SD	Mean importance attributed	SD
Familiarity with organ donation	6.70	0.7	6.30	1.19	3.56	0.5	3.44	0.5
Familiarity with choice registration	6.78	0.59	6.11	1.3	3.57	0.5	3.39	0.49

**Table 5:** Choice about donation registration divided by group of belonging.

Group of belonging	Choice registered		Choice not registered	
	*N*	%	N	%
Local healthcare professionals	28	51.9	26	48.1
Critical area healthcare professionals	34	60.7	22	39.3
Young adult population	23	41.8	32	58.2
Adult population	24	43.6	31	56.4
Opinion leaders	10	38.5	16	61.5
Registry office employees	22	47.8	24	52.2
Hospital healthcare professionals	37	67.3	18	32.7

### 3.4 Testing the TPB model

The linear regression test with regard to attitude, showed that the TPB model adequately explains its variability (*p* < 0.001, *R*^2^ = 0.354). Between the variables taken into consideration (having thought about donation, group to which the respondents belong, gender, being registered with an association, age, education, familiarity with donation and/or with consent registration), those showing a significant influence in the model are familiarity with the topic of donation (*p* = 0.04), familiarity with the topic of consent to donation registration (*p* = 0.002) and having already thought about donation (*p* < 0.001). In this model, the association between attitude and intention to donate was very strong (p < 0.001). In fact, the attitude showed by participants predicted their intention to register their consent with 93.1% accuracy. The *odds ratio* between the two variables is 3:1. Consequently, a more positive attitude increases by 3 to 1 the probability that an individual intends to register his or her consent to donation.

**Figure 2:**
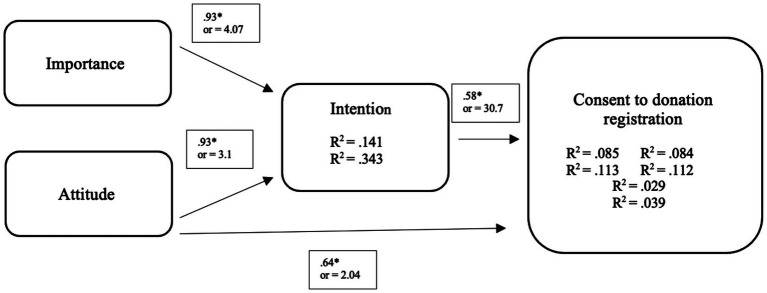
Logistic regression model between importance, attitude, intention and consent to donation registration.

The regression analysis showed that the association between intention and effective consent registration was significant (*p* = 0.001), showing that the intention to register one’s consent can be predicted with 14.8% accuracy when people did not give their consent, and 99.4% accuracy when they registered their consent, with an *odds ratio* of 30:7. The regression between attitude and consent registration was significant (*p* < 0.001), showing that attitude predicted 63.8% of variability in terms of consent registration with an *odds ratio* of 2:04. Finally, a significant relationship between importance and intention to donate was found (*p* = 0.006), predicting the intention with 92.8% accuracy (*odds ratio* = 4:07). See Figure 2 for details.

In the present regression model demographic variables were also tested. However, they did not show a significant influence, apart from attitude (*p* < 0.001). Looking at the *odds ratio,* it is possible to see how certain variables increase the possibility of the person to have registered their own consent (see Table 6). For instance, having reached a post-lauream level of education increases the probability by 2:14 times when compared to having a middle school diploma. Being a male, on the other hand, decreased the probability by 8%.

**Table 6:** Logistic regression model on consent registration.

Variables	*Β*	Significance	Odds ratio
Attitude	0.625	0.000	1.869
Importance	0.308	0.274	1.361
Age	0.010	0.431	1.010
Gender (M)	−0.075	0.764	0.927
Middle School Diploma		0.091	
High School Diploma	0.685	0.265	1.983
Bachelor’s Degree	0.106	0.864	1.112
Post-lauream	0.761	0.242	2.140
Local healthcare professionals		0.349	
Critical area healthcare professionals	0.380	0.378	1.463
Young adults	0.060	0.911	1.061
Adults	0.129	0.793	1.138
Opinion leaders	−0.516	0.354	0.597
Registry office employees	0.236	0.622	1.266
Hospital healthcare professionals		0.076	2.193

### 3.5 What is the role of participating in the focus group?

By measuring attitude, importance and consent registration intention before and after the focus group, it was possible to assess if these variables changed as a result of participation in the group.

Attitude, importance and consent registration intention were measured before and after the focus group, to assess variations (see Table 7). The results showed that attitude (*Z* = −4.06, *p* < 0.001) and importance of donation (*Z* = −4.62, *p* < 0.001) significantly increased after the focus group, whereas intention to register one’s consent did not (*p* = 0.125).

**Table 7:** Before and after measurements of attitude, importance and intention to register one’s consent.

Variables	Before the focus group	After the focus group
Attitude	6.45 (SD = 1.05)	6.56 (SD = 0.99)
Importance	3.48 (SD = 0.5)	3.58 (SD = 0.49)
Intention to register one’s consent	89.2%	94.8%

After the focus group, participants were asked to evaluate the experience of participating, by responding to 9 items on a Likert scale ranging from 1 to 7. Since the correlation between the items was very high (*α* = 0.93), the authors summed the items in the form of a general satisfaction rating, with a mean score of 6.38 (SD = 0.73). This index was positively correlated to the mean attitude (*p* < 0.001) and the attributed importance measured before the focus group (*p* = 0.001) and the intention to register one’s consent to donation (*U* = 1721.5, *p* = 0.039) measured after the focus group. This index, on the other hand, did not correlate with the before and after increase in attitude (*U* = 6,066, *p* = 0.976) nor the importance attributed to donation (*U* = 1721.5, *p* = 0.039), underlining that those who were more satisfied with the participation to the focus group did not show a greater increase in their attitude, nor with regard to importance attributed to donation after participating.

## 4 Discussion

The results of the study reveal interesting data regarding attitude, importance of donation and decision of registering one’s consent in different groups of the Italian population, providing us with useful elements to discuss and reflect on potential implications and future directions regarding this topic in Italy.

Our results indicate a significant association between gender and attitude, but not with importance and effective consent to donation registration. In line with the CNT report (Centro Nazionale Trapianti, 2021), this finding confirms that gender differences persist in attitudes, although they do not translate into higher registration rates. In contrast, unlike what has been reported in the literature (Falomir-Pichastor et al., 2011), it has not been found that age was significantly related to attitude, nor with the decision to register one’s consent. However, it was significantly associated with the importance attributed to donation. This partially confirms previous evidence (Falomir-Pichastor et al., 2011), while also highlighting a possible specific pattern in our population. Furthermore, in line with the literature (Falomir-Pichastor et al., 2011), a higher level of education corresponded to both a more favorable attitude and a higher importance attributed to donation. Although not significant, in our sample those with a higher education were more likely to have already registered their consent.

Another important result of our study relates to the identification of differences in attitude towards donation and consent registration between groups, especially between healthcare professionals (local healthcare professionals, critical area healthcare professionals, and hospital healthcare professionals) and the general population (registry office employees, adult population, young adult population and opinion leaders). Our data show that healthcare professionals expressed a more positive attitude, importance and a higher rate of effective consent registration than other groups. These findings are consistent with studies conducted with healthcare professionals and students in Italy (Fontana et al., 2017; Terraneo and Caserini, 2022) and abroad (Elsafi et al., 2017; Hakeem et al., 2021), confirming that greater exposure to the topic generally corresponds to more favorable positions. However, as already noted in the literature (Fontana et al., 2017; Terraneo and Caserini, 2022; Elsafi et al., 2017; Hakeem et al., 2021), a discrepancy remains between positive attitude and effective registration. Moreover, different studies have shown that misinformation and misbeliefs about brain death and organ donation are widespread even among healthcare professionals (Babaie et al., 2015; Bøgh and Madsen, 2005; Lomero et al., 2017), and only a small proportion feel adequately informed to register a conscious choice (Terraneo and Caserini, 2022).

In our study, in terms of the groups of healthcare professionals, the one that showed a more positive attitude was the healthcare professionals of critical areas, that is the group directly involved in the donation process (both organ procurement and transplantation), underlining how direct exposure and involvement, together with a reported higher number of individuals knowing someone who donated/received an organ and who registered their consent, positively influenced their attitude. Interestingly those showing the least positive attitude were the groups of the adult population and registry office employees.

The literature points out that several factors can negatively influence the decision of becoming a donor, such as fear of having the body “ruined” (Miller et al., 2020), religious beliefs (Lauri, 2009), misleading knowledge about brain death, and lack of trust in the healthcare system (Skowronski et al., 2020). Exploring the attitude and experience of registry office employees is of vital importance as they play a fundamental role in querying and registering one’s consent to donation in Italy, given that they collected 86.6% of the total of registrations in 2021 (Centro Nazionale Trapianti, 2022), and could play a role in the decision with regard to registering one’s choice on donation, as well as in choosing consent or opposition.

Generally, the literature shows that being informed about organ donation is associated with a positive attitude (Mostafa, 2008) as well when one shares and discusses this topic with one’s family (Stadlbauer et al., 2020). Unfortunately, to date, there is still a widespread lack of information (Terraneo and Caserini, 2022) or reported misbeliefs with regard to the topic, even in highly educated populations (Arisal and Atalar, 2019). In this sense, one of the most debated and divisive issue is regarding the definition and understanding of brain death, which has been found not to be understandable and acceptable as a “real death condition” in the general population (Othman et al., 2020), even more evidently in adolescents (Stadlbauer et al., 2020) and in healthcare professionals (Babaie et al., 2015; Bøgh and Madsen, 2005).

Our results show that not only being familiar with the topic in the sense of knowing someone who has donated or received an organ was associated with a more positive attitude, but also knowing someone who had registered their consent to post-mortem donation. This is consistent with previous literature (Falomir-Pichastor et al., 2011; Stadlbauer et al., 2020), which emphasizes the role of familiarity and interpersonal discussion in fostering knowledge and positive attitudes. Interestingly, those registered with at least one association with regard to donation reported a significant higher rate of consent to donation registration, but not a significantly more positive attitude, a result that is in line with previous findings (Hyde et al., 2013) reporting that those who were registered with a blood donation association were more likely to donate their organs, show prosocial behaviors and an altruistic identity (Hyde et al., 2013) and exhibit social responsibility (Falomir-Pichastor et al., 2011).

Studying the relationship between attitude, intention and behavior is of great interest with regard to the topic of organ donation in the field of social and health psychology. The Theory of Planned Behavior (TPB) (Ajzen, 1991) affirms that intention is the construct that most directly predicts behavior, being directly influenced by attitude. Our results confirm this model, as both the relationship between attitude and intention and the one between intention and behavior were significant. Furthermore, taking into consideration demographic variables such as age, education, gender, group of belonging, and contextual aspects such as the familiarity with the topic and having thought about that, together with attitude and importance of donation, the predictiveness of the regression model acquires greater value. This underlines the importance of developing a complex model to explain more accurately the decision to register one’s choice regarding donation.

Last but not least, our results showed a significant increase in attitude towards post-mortem organ donation when comparing the before and after of participation in the study, specifically with regard to the focus group discussions around the topic. This effect was observed independently of satisfaction with the experience and is consistent with studies showing that opportunities for discussion can foster changes in attitudes (Siegel et al., 2010; Kuhar et al., 2019).

Considering our results, to support individuals in making a conscious choice on post-mortem donation, and to fill the gap that exists between positive attitude and effective consent registration, it is fundamental to work conjunctly on different levels, such as in terms of information, education, sensibilization and the creation of tailored awareness campaigns discussing medical, ethical, legal, psychological and sociological aspects (Terraneo and Caserini, 2022). Fostering knowledge and solving misbeliefs around post-mortem organ donation is of great importance, not only in the general population, but also in the case of healthcare professionals. As shown by our results, providing time and space for open discussion with peers, followed by an eventual educational discussion with clinical experts aimed at answering all the groups’ doubts and questions, could foster the process of consent to donation registration. This underlines the importance of developing and implementing awareness campaigns that give space and attention to testimonials, making it possible for individuals to meet and discuss the topic with people who have been involved in this experience, such as donor’s family members (Rumsey et al., 2003).

Last but not least, it is urgent to provide to opinion leaders and people directly involved in the process of consent registration with an accurate and complete education in order to support them in their role of providing unbiased (both positive or negative) information when dealing with and supporting individuals in registering their choice about donation. In addition, it is urgent to increase the possibilities when it comes to registering one’s choice about post-mortem organ donation, as for many the non-registration is attributed to a lack of time and opportunity (Terraneo and Caserini, 2022).

## 5 Limitations

The sample of our study was composed of participants who already showed a positive attitude towards donation, reported a high level of education (greater than the Italian average) and had an awareness of the topic, as numerous of the participants were already registered with associations dealing with donation. This probably resulted from the active role of the Regional Transplant Centers in the recruitment of local participants for the study, that inevitably produced participants who showed awareness about the topic. However, it was possible to observe and test pre-post changes in individuals’ attitude towards post-mortem organ donation, the importance of donation and the intention to register one’s consent.

## 6 Conclusion

Little research has been carried out in Italy to investigate attitude towards post-mortem organ donation in different groups of the Italian population, apart from healthcare professionals and students. In fact, this is the first study to involve different groups of the population involved in the process of consent to donation registration.

Our results underline the influence of various factors associated with predicting the behavior of individuals in terms of becoming a donor, such as age, education, gender and group of belonging, familiarity with the topic, in addition to attitude and value. This points out the need to make use of a multi-variable model to explain more accurately how individuals arrive at the decision to register one’s consent regarding donation.

To conclude, our findings will provide experts and policy makers with important information about the potential future direction with regard to awareness raising and informational campaigns to guide citizens at arriving at an informed and conscious choice regarding post-mortem donation, and in the exploration of different populations involved and playing a significant role in this decision-making process.

## Data Availability

The datasets presented in this article are not readily available due to the nature of this research and participants of this study did not agree for their data to be shared publicly. Requests to access the datasets should be directed to sabrina.cipolletta@gmail.com.
